# Computational modelling of the crushing of carbon fibre-reinforced polymer composites

**DOI:** 10.1098/rsta.2021.0336

**Published:** 2022-09-19

**Authors:** Brian G. Falzon

**Affiliations:** ^1^ School of Engineering, RMIT University, GPO Box 2476, Melbourne, Victoria 3001, Australia; ^2^ Advanced Composites Research Group, School of Mechanical and Aerospace Engineering, Queen's University Belfast, BelfastBT9 5AH, UK

**Keywords:** composite materials, finite-element modelling, computational simulation, crashworthiness

## Abstract

The use of lightweight carbon fibre-reinforced polymer (CFRP) composites in transportation vehicles has necessitated the need to guarantee that these new materials and their structures are able to deliver a sufficient level of crashworthiness to ensure passenger safety. Unlike their metallic counterparts, which absorb energy primarily through plastic deformation, CFRPs absorb energy through a complex interaction of damage mechanisms involving matrix (polymer) cracking, fibre/matrix debonding, fibre pull-out/kinking/fracture, delamination and inter/intralaminar friction. CFRP is primarily deployed as a laminate and can potentially deliver a higher specific energy absorption than metals. Translating this capability to a structural scale requires careful design and is dependent on geometry, fibre architecture, laminate stacking sequence and damage initiation strategies for optimal uniform crushing. Consequently, the design of crashworthy CFRP structures currently entails extensive physical testing which is expensive and time consuming. This paper reports on progress and challenges in the development of a finite-element computational capability for simulating the crushing of composites for crashworthiness assessments, with the aim of reducing the burden of physical testing. It addresses the ‘tyranny of scales’ in modelling structures constructed of CFRP composites. Intrinsic to this capability is the acquisition of reliable material data for the damage model, in particular interlaminar and intralaminar fracture toughness values. While quasi-static values can be obtained with a reasonable level of confidence, results achieved through dynamic testing are still the subject of debate and the relationship between fracture toughness and strain rate has yet to be satisfactorily resolved.

This article is part of the theme issue ‘Nanocracks in nature and industry’.

## 1. Introduction

Carbon fibre-reinforced polymer (CFRP) composites are increasingly being used in the development of lightweight transportation structures, in a concerted effort to minimize their environmental operational footprint. Ambitious national and intergovernmental agreements to reduce harmful emissions are likely to continue to exert pressure on this industry sector to move towards net-zero emissions transportation. The aerospace industry has been at the forefront of exploiting lightweight materials in airframe construction, and approximately half the structural weight of the latest generation of Airbus and Boeing wide-body passenger aircraft is made of CFRP. This has resulted in a 20% reduction in structural weight compared with similar metallic aircraft, with a commensurate decrease in fuel consumption. Several high-performance road vehicles, racing cars and premium electric vehicles also have a predominantly CFRP monocoque structure.

**Figure 1.**
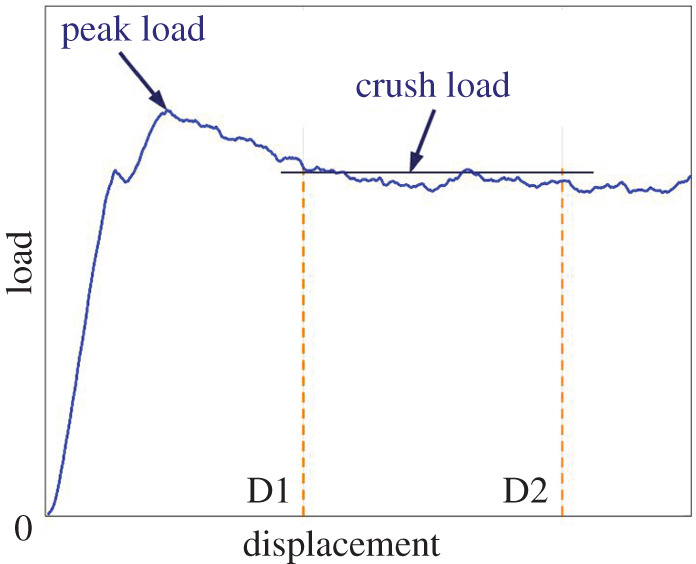
Load–displacement curve for progressive crushing. (Online version in colour.)

The evolution of composite transportation structures has been based on the gradual substitution of metallics with CFRP. The capacity to absorb a certain amount of energy in a crash event is expected of transport vehicles. The crashworthiness of these structures is predicated on their ability to (i) minimize harmful deceleration forces transmitted to the occupants, (ii) maintain a survivable cabin volume, (iii) allow evacuation paths and (iv) retain items of significant mass [1]. In essence, the structural requirements for crashworthiness may be represented on a load–displacement curve. With reference to figure 1, a structure undergoing progressive crushing should not induce a high initial peak load which could cause injury to occupants. Ideally, a uniform ‘crush stress’ (crush load/cross-sectional area) is subsequently maintained over a crush distance or stroke (D2–D1) such that the energy absorbed (area under the curve) is maximized. Jacob *et al*. [2] also pointed out that the rate of energy absorption is important where the longer the time period, the more crashworthy the structure.

Crashworthy metallic structures absorb energy primarily by plastic deformation. Tubular metallic structures have been extensively used in the design of automotive energy absorbers, where they are shown to be highly efficient in absorbing energy by a process of plastic folding (e.g. [3,4]). CFRP composites do not exhibit substantial plastic deformation and energy absorption is achieved through an entirely different process involving a complex interaction of matrix and fibre fracture, fibre/matrix interfacial debonding, fibre pull-out, fibre kinking, delamination and inter/intralaminar friction. The lay-up and fibre architecture also have a significant influence on the crushing response. As a result, the ability of CFRP structures to absorb energy in a crash scenario has been the subject of considerable interest (e.g. [2,5–16]). CFRP crash structures are also more sensitive to initial crush conditions such that an appropriate ‘trigger’ needs to be considered to initiate a failure process that maximizes energy absorption [17,18]. It is apparent that the increase in the number of parameters which govern the energy absorbing capacity of CFRP structures suggests that greater design effort is required compared with their metallicequivalent.

Indeed, this additional effort applies to the use of CFRP in general. This is compounded by limited in-service experience of composite materials, which in the case of the development of composite passenger aircraft has compelled the certification authorities to take a cautious approach to certification. Consequently, certification of composite aerostructures is a costly undertaking which currently requires extensive experimental testing programmes, often supported by simulation, executed through a building block approach represented as a test pyramid [19]. The bottom of the pyramid represents the testing of thousands of generic coupons for material characterization, progressing through to more complex non-generic structural details and assemblies.

Remarkably, there are currently no airframe-level crashworthiness certification requirements beyond seat-level ones (with the exception of military rotorcraft [20]), where it is presumed that the airframe provides an acceptable level of crashworthiness. There is an implicit assumption at the core of these certification requirements that airframes are predominantly of metallic construction. In 2018, a working group was established by the Federal Aviation Administration (FAA) to examine the adequacy of current FAA certification requirements in the crashworthiness of transport aircraft [21]. Group members representing regulatory authorities and research communities advocated for additional certification requirements when composites were used. This was resisted by manufacturers who argued that it would inflate development costs and deliver a negligible improvement in safety. Crashworthiness of the current generation of composite passenger aircraft was demonstrated by addressing special conditions imposed by the certification authorities which obliged the manufacturer to ensure that the level of crashworthiness was comparable with their metallic counterpart. This comparative assessment was delivered through simulation supported by physical testing. In essence, the working group's recommendation would see the adoption and extension of these special conditions as part of the FAA's standard certification requirements for non-metallic fuselages.

It is encouraging to note that well-designed CFRP crush structures have been shown to deliver higher specific energy absorption then metallic structures [22] but additional care needs to be taken in their design. The debate surrounding the requirement for additional certification testing in the crashworthiness assessment of aircraft is likely to persist. The emergence of electrical vertical take-off and landing (eVTOL) urban air mobility vehicles could see a proliferation of flying vehicles where even within a highly regulated airspace and sophisticated autonomous control systems, crashes can be expected [23,24].

Within the automotive industry, it is observed that ‘all-composite’ monocoque road vehicle bodies tend to be underpinned by metallic crush structures owing to the challenges faced in the cost-effective design of composite crush elements. Replacing these metallic components with crashworthy composite ones is an area of ongoing research and development [25,26]. Unlike the aerospace industry, there is greater variability in the minimum level of automotive safety mandated in different countries. The US New Car Assessment Program (NCAP) and its European counterpart (EuroNCAP) were created to independently rate the safety of vehicles against national minimum standards. While not having certification or regulatory authority, these programmes have spurred on manufacturers to enhance their safety features, not least their passive crashworthiness [20]. The move towards electric vehicles has added impetus towards the adoption of ever lighter structural materials to mitigate the higher weight of the electrical powertrain compared with one based on an internal combustion engine. Hence there is likely to be further uptake of composites in mass-produced vehicles as the cost of the material reduces and manufacture speeds increase.

The extensive physical testing undertaken in the development of composite transportation structures; in particular, the test programmes required for the certification of composite airframes is costly and time consuming. This has driven the pursuit of reliable and accurate computational modelling to support the certification process, with the primary objective of reducing the extent of physical testing and consequently, cost. A computational methodology which can capture the crushing of a composite structure involves the implementation of a damage model. Several damage models, with different levels of fidelity, have been proposed over recent years, primarily for assessing the damage arising from low-velocity impact [27]. Additional computational strategies need to be implemented to create a reliable computational tool to model composite crushing for crashworthiness assessments.

In this paper, a brief overview of a finite-element-based high-fidelity damage model is presented, using fracture mechanics and continuum damage mechanics (CDM), to capture interlaminar and intralaminar damage, respectively. This model was originally developed for modelling low-velocity impact events and assessing residual strength, and subsequently adapted to deal with crushing. Its potential use in a multiscale framework is also discussed. Such high-fidelity models typically require fracture toughness data and strategies and shortcomings in their acquisition is reviewed.

## 2. Computational damage model

### (a) From nanocracks to macroscale crushing: the tyranny of scales

For a conventional laminated fibre-reinforced composite structure, damage may be distinguished by that which occurs between plies, i.e. interlaminar delamination, and that which occurs within a ply, i.e. intralaminar damage. The former is predominantly matrix dominated, while the latter may be either matrix or fibre dominated, depending on the load state, and where the fibre–matrix interface plays a critical role in the composite's response. In developing a computational model, a choice needs to be made regarding the scale at which an analysis is to be executed. By definition, the constituents of the composite exist in distinct phases, e.g. carbon fibre and matrix, and a damage model may be developed at (i) the nano/microscale level, where the fibres and matrix are distinctly modelled and damage initiation is modelled at the sub-micron/nanoscale; (ii) the mesoscale level where the properties are homogenized at the ply level; or (iii) the macroscale level where the properties are homogenized at the structural scale. The choice of scale will have a profound influence on the input data required for the computational model, and the means of acquiring these. Within a finite-element context, it also dictates the type of analysis that can be undertaken within a reasonable time frame and available computational resources. As pointed out by Fish *et al*. [28], physical properties at the finer scales are believed to be better understood, but while capturing damage at the nano/microscale may give valuable insight into the initiation and evolution of damage it is not immediately amenable for use in the modelling of engineering structures which are likely to be orders of magnitude larger in scale. Conversely, a macroscale damage model may not be able to capture damage events that occur at lower scales, which eventually propagate, or coalesce, and lead to catastrophic failure.

The linking of scales in the modelling of materials and structures has attracted considerable debate. Batterman [29] explored what he interpreted as a dichotomy which has developed within the modelling community, where one side subscribes to the view that fundamental bottom-up approaches are the most accurate, from which all observed phenomena can be deduced, while the other believes that top-down continuum type modelling strategies are superior. It is evident that both strategies are required but the means of linking these remains a challenge. Fish *et al*. [28] propose the use of machine learning for linking scales and Batterman [29] suggests that consideration of mesoscale structures ‘provide the bridges that allow us to model across scales'.

A compromise between these two scale extremes is to adopt a mesoscale damage model where the properties are homogenized at the ply level. Modelling realistic engineering structures at this scale will still require considerable computational resources which may be impractical within industry. The use of a multiscale approach, where information from a lower scale analysis informs a higher scale model, is a viable strategy for reducing the computational effort. One such multiscale approach, appropriate for crushing analysis, is discussed briefly through an example in §4c.

### (b) Explicit dynamic analysis

The highly nonlinear behaviour associated with the modelling of damage and crushing of composites requires a robust computational scheme. Various solution schemes are available for solving the system of nonlinear equations in a finite-element formulation, each presenting advantages and limitations [30]. It is well established that explicit dynamic finite-element analysis offers a high degree of numerical stability. It is based on solving the equation of motion in a ‘time-marching’ fashion over small time steps, making this approach ‘conditionally stable’ where the time step must be kept below a critical value, of the order of the time an elastic stress wave takes to propagate across the shortest dimension of the smallest element in a finite-element mesh. While this method is very robust, these time steps can become very small in the analysis of composite damage, thereby requiring long execution times. Various techniques are available for artificially speeding up the solution time. One approach is to use ‘mass scaling’. By the definition of the critical time step given above, it may be easily shown that this is proportional to the square root of the density of the material. For a given finite-element volume, increasing the mass translates to an increase in density which will also increase the size of the critical time step. Ideally, the solution process should be capable of ‘selective mass scaling’ where only specific elements are scaled. Commercially available explicit finite-element systems usually offer this capability. Another strategy is to increase the loading rate. In either case, these strategies need to be used with care to ensure that inertial effects, which are artificially added to the model, are not significant and the associated kinetic energy is a small fraction of the overall internal energy of the model. Where the material is also strain rate dependent, increasing the loading rate may also adversely affect the quality of the solution.

### (c) Interlaminar damage

**Figure 2.**
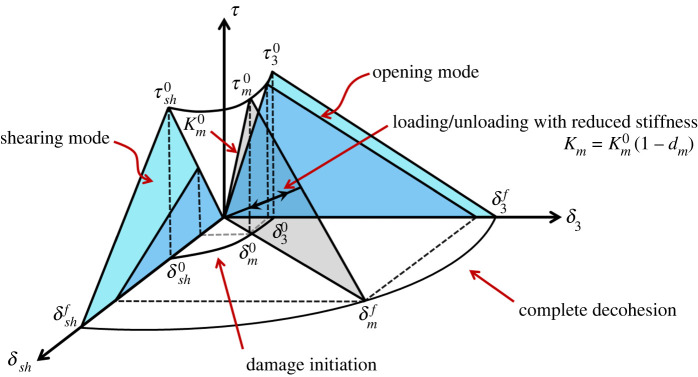
Mixed-mode delamination model. (Online version in colour.)

At the mesoscale, interlaminar damage is typically modelled using a cohesive element which may be formulated with a finite or zero thickness. It is inserted at the interface of finite-elements where delamination may be expected, and a constitutive law is defined which governs the separation of this interface, to simulate delamination. This approach was first introduced by Hillerborg *et al*. [31] in their work on the modelling of crack growth in concrete and builds on concepts introduced by Barenblatt [32] and Dugdale [33]. Separation of the two surfaces defining the interface is governed by a mixed-mode interaction between the opening mode (mode I) and a shearing mode (mode II). A bilinear ‘traction-separation’ law is often used to represent the individual modes where the response for each mode is defined by a critical strength ($\tau_{3}^{0}$—mode I, $\tau_{\text{sh}}^{0}$—mode II), associated critical separation ($\delta_{3}^{0}$—mode I, $\delta_{\text{sh}}^{0}$—mode II) and a critical maximum separation ($\delta_{3}^{f}$—mode I, $\delta_{\text{sh}}^{f}$—mode II), figure 2. The formulation must also ensure that there is no interpenetration of the interface under compression loading (closing mode) and allow for shear reversal. The initial stiffness, $K_{m}^{0}$, must be high enough to ensure that the compliance of the overall structure is not significantly changed but not so high that it leads to numerical difficulties. Various approaches for determining $K_{m}^{0}$, proposed in [34–37], yield similar values.

A quadratic damage initiation function has been shown to be suitable for predicting mixed-mode delamination onset, $\tau_{m}^{0}$ (and $\delta_{m}^{0}$) [38]. This is followed by damage propagation represented by linear softening behaviour for $\delta_{m}^{0}<\delta <\delta_{m}^{f}$ where the area under the constitutive curve is a measure of the fracture energy, and the reduction in stiffness is defined by a monotonically increasing internal damage variable, $0_{\delta_{m}^{0}}\leq d_{m}\leq 1_{\delta_{m}^{f}}$, where $K_{m}=K_{m}^{0}(1-d_{m})$. Several mixed-mode damage propagation criteria have been proposed including a power law criterion [39] and a semi-empirical criterion [40]. The appropriateness of a bilinear constitutive softening law has been explored by several researchers. Other forms have been proposed, e.g. [41], but it has been repeatedly demonstrated that the shape of this softening law has little influence on the accuracy of capturing delamination as long as the fracture energy (the area under the constitute curve) is accurate and the critical strengths approximate the strengths of the material [42,43]. It has been observed that under very high loading rates, the abrupt gradient change at the apex of the bilinear curve (critical strength point) and at the maximum displacement (complete decohesion) may lead to numerical instabilities in an explicit analysis. In [44], it was shown that high stress waves may be generated at these points which induce high-frequency vibrations which break interface elements in the vicinity. This may be mitigated through the implementation of numerical damping strategies or by using a constitutive curve which is a smooth function such as a polynomial of appropriate order. Interface elements have also been used at the microscale level to model the fibre–matrix interface in a representative volume element (RVE) [45,46] and at the mesoscale level to model intralaminar fibre splitting under tensile or shearloading [47,48].

The length of the cohesive (or process) zone ahead of the crack tip will dictate the required mesh density since the method requires a mesh which is fine enough to capture the variations in traction ahead of the crack tip. Turon *et al*. [49] undertook an extensive investigation of the material, geometric and loading parameters which influence the length of the process zone. CFRP composites were shown to have a process zone of the order of a millimetre which consequently requires sub-millimetre interface elements. This may prove impractical in large-scale applications. Work by the same research group showed that by reducing the maximum strengths defined in a bilinear constitutive law, e.g. $\tau_{3}^{0}$ and $\tau_{\text{sh}}^{0}$in figure 2, but maintaining the correct fracture energy dissipation per unit area of crack surface, the process zone is extended over a greater number of elements permitting the use of a courser mesh than may otherwise be possible [37]. Such an approach needs to be used with caution as the lower the strength, the less accurate is the stress state near the crack tip.

### (d) Intralaminar damage

The use of CDM in the analysis of intralaminar damage in laminated composites has garnered much support within the modelling community with a focus on capturing impact damage (e.g. [50–57]) and predicting compression after impact (CAI) strength (e.g. [54,55,58,59]). CDM is based on the concept proposed by Kachanov [60] and further developed by Lemaître & Chaboche [61]. Using a one-dimensional element example, under loading, microscopic cracks and voids form within a representative volume of material, leading to a reduction in the effective load bearing area, from the original cross-sectional area, *A*, to $\tilde{A}$. The reduction of this load bearing area provides a measure of damage within the RVE, $\tilde{A}/A=(1-d)$, where the damage parameter, *d*, similarly to the definition given for the softening law association with interlaminar damage, is bounded (0≤d≤1) and monotonically increasing to prevent non-physical healing. By conceptualizing $\tilde{A}$ as the cross-section area of a ‘fictitious’ undamaged material under the same loading, we can relate the transmitted stress, σ, to the ‘effective’ stress, $\tilde{\sigma }$, in the fictitious undamaged configuration, $\sigma =\tilde{\sigma }(1-d)$. By assuming that the same strain is acting on the damaged and fictitious pristine material, it may be shown that this leads to a linear reduction in modulus, $E=\tilde{E}(1-d)$.

**Figure 3.**
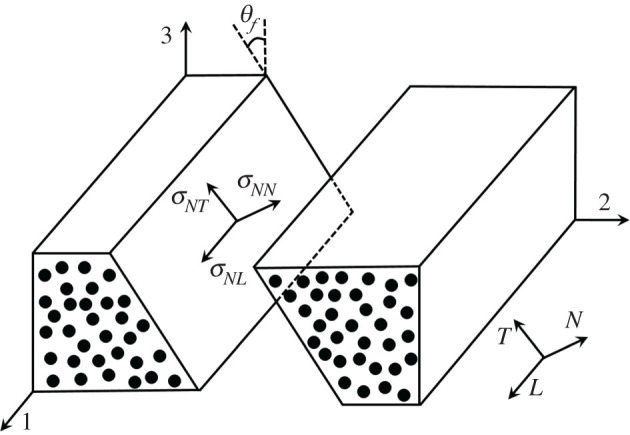
Coordinate systems for a representative volume element (RVE) of a unidirectional (UD) composite.

A constitutive plane stress model for anisotropic damage in fibre-reinforced composites was first presented in [62] and has formed the basis for the numerous formulations that have since been proposed. In the approach developed by the author, a RVE of a homogenized fibre-reinforced polymer composite is subjected to a full three-dimensional stress state. It is assumed that the fibres are unidirectional along the 1*-*direction with directions 2 and 3, representing the transverse and through-thickness directions, respectively, figure 3. This may be assumed to be the material coordinate system. Two intralaminar internal damage variables are defined, one to represented fibre-dominated failure modes, *d*_fib_, and the other to capture matrix-dominated failure modes, *d*_mat_.

#### (i) Fibre-dominated failure modes

**Figure 4.**
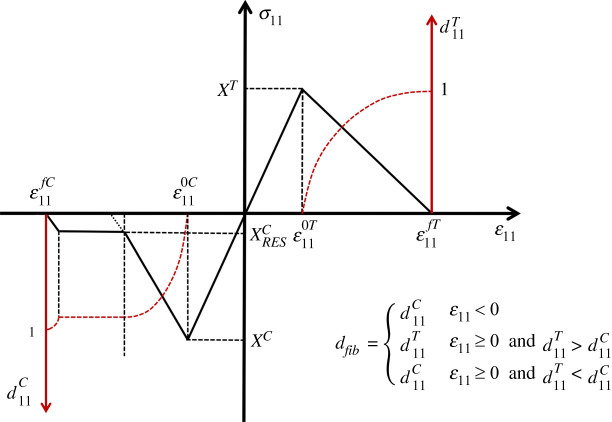
Bilinear constitutive law for tensile/compressive longitudinal loading. (Online version in colour.)

A bilinear law analogous to the one used to represent interlaminar damage, has been shown to be adequate in capturing fibre-dominated damage in tension [5]. In compression, where damage is initiated by fibre kinking arising from inevitable fibre misalignment [63], this law is modified to account for the residual strength arising from entrapped debris [64], figure 4. Unlike the case for the constitutive law used for interlaminar damage, the use of a volume element requires that the intralaminar bilinear law is given as a stress–strain relation where the damage manifests as a degradation in modulus and the area under the curve is a measure of strain energy density. This volumetric energy is related to the fracture energy (critical energy release rate) of the material, a measurable quantity, through a characteristic length, $l_{\text{fib}}$, where the volume of material divided by this characteristic length, yields the fracture surface area. The introduction of a characteristic length also ensures mesh objectivity and limits on the maximum characteristic length are also imposed to ensure material stability [65]. Under pure tension/compression, the fracture plane is assumed to be perpendicular to the fibre direction. Damage initiation, in either tension (*T*) or compression (*C*), is typically given as a function of critical tensile/compressive strengths and may include a modifying shear term as proposed in [66]. Issues arising from the use of modifying loads are discussed in [67]. $d_{\text{fib}}$ is a monotonically increasing function which is based on two internal damage variables, one which tracks damage arising from tensile loading parallel to the fibres, $d_{11}^{T}$, and the other for compressive damage, $d_{11}^{C}$ [68].

#### (ii) Matrix-dominated failure modes

Under a general state of three-dimensional stress acting on a RVE, transverse (to the fibre direction) stresses and/or shear stresses may lead to a fracture plane at any permissible orientation with its surface vector perpendicular to the fibre direction. Experimental observations of unidirectional (UD) composite specimens subjected to uniaxial transverse compression, have shown fracture angles of $\approx 53^{\circ }$ [69] to $\approx 56^{\circ }$ [70] and as pointed out in [67] suggests that failure is governed by a combination of yielding and Mohr–Coulomb friction where a transverse compressive stress impedes fracture along an ‘action’ plane acted upon by shear stresses, as shown in figure 3. Puck & Shurmann [71,72] proposed damage initiation functions which account for Mohr–Coulomb friction when under compressive transverse stress. Further modifications where proposed by Camanho *et al*. [73] and Catalanotti *et al*. [74] to account for *in situ* strengths. Having established a formulation for damage initiation there remains the need to determine the orientation of the fracture plane. In the model developed by the author and co-workers, this fracture plane orientation is determined at the onset of damage within each finite-element and remains fixed with subsequent loading. Brent's search algorithm [75] is used to search for the orientation which maximizes the damage initiation functions. The procedure for calculating the area of the fracture plane is given in [76] and dividing the element volume by this area yields the characteristic length, $l_{\text{mat}}$.

Intralaminar matrix plasticity plays a crucial role in the evolution of damage and leads to permanent macroscale deformations, such as indentation due to localized impact events, which may significantly influence a structure's residual strength [77]. This plasticity is driven by the distortional strain energy associated with shear stress components, $\gamma_{ij}$, expressed as the sum of elastic, $\gamma_{ij,\text{el}}$ and inelastic/plastic, $\gamma_{ij,i\text{n}}$, components, where i,j=1,2,3,i≠j. A nonlinear constitutive relation for each shear stress component was expressed in [78] which accounts for isotropic strain hardening for loading below a critical load threshold. This threshold is defined such that unloading at this point, would lead to a reduced secant shear modulus with its origin at the permanent plastic strain, and corresponds to the critical stress associated with elastic CDM. It follows that we may define each corresponding damage parameter, $d_{ij}^{\;}$ as the sum of a damage parameter associated with strain hardening, $d_{ij}^{I}$, i.e. pre-peak damage, and another with damage arising from strain softening, $d_{ij}^{II}.$ This approach assumes that some stiffness reduction occurs prior to reaching the critical load.

**Figure 5.**
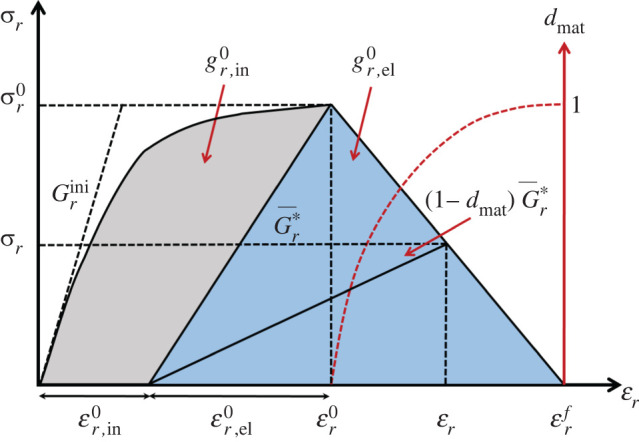
Constitutive law for matrix-dominated damage. (Online version in colour.)

Matrix damage on a fracture plane is primarily determined by loading in shear and modified by transverse tension/compression. With reference to figure 3 and figure 5, we may use the$l^{2}$-norm of the vectors of stress, $\sigma_{r}=\sqrt{{\sigma_{NN}}^{2}+{\sigma_{NT}}^{2}+{\sigma_{NL}}^{2}}$, and corresponding strain, $\varepsilon_{r}=\varepsilon_{r,\text{el}}+\varepsilon_{r,\text{in}}$, where $\varepsilon_{r,\text{el}}=\sqrt{{\varepsilon_{NN}}^{2}+\gamma {{\;}_{NT}^{\text{el}}}^{2}+\gamma {{\;}_{NL}^{\text{el}}}^{2}},\varepsilon_{r,\text{in}}=\sqrt{\gamma {{\;}_{NT}^{\text{in}}}^{2}+\gamma {{\;}_{NL}^{\text{in}}}^{2}}$, acting on the fracture plane, to derive an expression for $d_{\text{mat}}$ with which the shear modulus, $G_{r}$, and shear stresses on this fracture plane may be degraded. The relationship between $d_{\text{mat}}$ and $d_{ij}$ is explained in [78]. It is worth noting that in this discourse, a material coordinate system has been assumed. A further transformation to a global coordinate system associated with a structural model is usually required. The corresponding critical energy release rate, associated with the shaded region in figure 5, is a function of the individual energy release rates associated with each stress component and is used to determine $\varepsilon_{r}^{f}$ [54]. A rigorous approach, originally proposed by Schapery [79] for describing the evolution of pre-peak damage using thermodynamically consistent work potential theory, was adopted in [56,80] for modelling impact damage on laminated composites.

### (e) Additional considerations for crush modelling

The interlaminar and intralaminar damage models, implemented within an explicit finite-element system, should be able to capture impact damage with a high degree of accuracy, provided the right material properties are supplied as input parameters (see §3) and contact logic is available to prevent interpenetration of elements. Contact conditions must also account for new surfaces that may form during the solution process. Often, the motivation for impact damage analysis is to determine the influence of such damage on structural integrity, i.e. residual strength. CAI strength is seen as a critical design driver and the ability to model this structural response has much in common with the ability to predict crush behaviour for crashworthiness assessments. The author and colleagues have reported a number of CAI validation case studies, e.g. [54,55], and will not be discussed here. To ensure computational robustness, certain additional measures need to be considered in the modelling of crush. While these are often also required to stabilize impact/CAI simulations, they become more critical in crush analysis.

#### (i) Spurious oscillations

In explicit dynamic analysis, spurious oscillations cannot be avoided due to the finite-element discretization, and they cannot be eliminated completely using a low-pass filter [81]. The use of a finite number of elements restricts the model's ability to capture high-frequency responses to wave propagation. Additional measures need to be taken to dissipate the energy associated with these spurious oscillations. An established approach is to add a viscous pressure term, commonly referred to as the bulk viscosity method, to the dynamic equilibrium equations. This pressure term has two components; a linear (with respect to volumetric strain rate) and a quadratic term. The linear term is used to damp the high frequency ‘ringing’ in elements under low pressures, while the quadratic term is used to dissipate energy associated with high-velocity gradients. The latter term is not usually required for the strain rates encountered during crushing. Other finite-element-based approaches to damping these spurious oscillations have been proposed, and a selection of these are discussed in [82].

#### (ii) Element distortion

Upon crushing, a finite-element may be forced to ‘invert’, leading to a negative volume, or undergo excessive shear distortion, which will cause the analysis to abort. Numerically, this can be detected by observing the sign of the determinant of the Jacobian matrix (det **J**) and mitigation measures invoked to prevent it from going negative. One strategy is to introduce another form of viscous damping at the nodes which restricts certain displacements leading to a negative Jacobian. This needs to be used judiciously as it may influence the quality of the solution. This form of distortion control is separate to that required for zero-energy modes associated with reduced-integration elements commonly used in explicit finite-element analysis. These zero-energy modes are often referred to as ‘hourglass modes’ because of the intrinsic shape formed by bilinear elements. The use of ‘hourglass control’ is an additional tool available to the analyst which imposes stiffness constraints and/or viscous forces to resist this form of deformation.

These measures have their limitations and there will be numerous instances in a typical crush simulation where element distortion cannot be adequately controlled. For such cases, element deletion needs to be considered. The evolution of damage within a finite-element may be such that its load carrying capacity may be partially preserved under specific load cases and greatly diminished in others. For example, an element which has undergone substantial matrix damage may still be capable of transmitting some loading in the fibre direction. Postponing deletion until all the internal damage variables are close to unity may result in deleterious distortions. The challenge is to remove these when as much useful work done by these elements is extracted prior to tripping the solution. Removing elements too early may affect the quality of the solution if a significant number of distorted elements are deleted prematurely.

As mentioned earlier, one approach is to track the sign and magnitude of the determinant of the Jacobian matrix and remove the element when this is close to zero. If the finite-element system does not provide such information as part of its solution database, this calculation will need to be executed as part of a user subroutine which will add to the solution time. Some finite-element systems do provide the determinant of the deformation gradient (det **F**), which is closely related, and this may be used instead. Det **F** yields the ratio of the deformed element volume to its original volume (cf. det **J** which compares the ratio of the deformed volume to its original volume in natural coordinates). A systematic study on the selection of limits on det **F** was undertaken in [83] and an element deletion strategy proposed where an element was deleted if det **F** limits were violated or the internal damage variable associated with fibre-dominated damage was close to unity.

#### (iii) Strain rate effects

An extensive database on the strain rate effects on the mechanical response of metals exists which has led to various strain rate dependent constitutive models being proposed over the past few decades [84] and adopted in various finite-element codes for crash simulation of metallic structures. By contrast, assessing the strain rate response of composite materials and their structures is a distinctly more complicated matter. There is a lack of consensus on the influence of strain rate on the crush behaviour of composite structures. For example, work by the author and his research group showed that composite cylinders, with a tulip trigger and a cross-ply UD carbon–fibre/epoxy lay-up, did not exhibit appreciable strain rate sensitivity over crushing speeds ranging from quasi-static to 100 s^−1^ [85]. By contrast, adopting a different carbon–fibre architecture (woven) and a chamfer trigger yielded a reduction in specific energy absorption (SEA) with strain rate [86], whereas an increase in SEA and peak force was recorded for the crushing of square tubes made from woven CFRP [87]. Chamfered open C-section woven and woven/UD hybrid CFRP specimens exhibited a reduction in SEA [88]. It is not the purpose of this paper to give an exhaustive overview of the literature reporting on strain rate investigations, but the few highlighted works serve to demonstrate that the rate sensitivity of a laminated composite structure is dependent on numerous factors, including the orientation and location of the plies within a laminate stack, the structure's/specimen's geometry, fibre architecture and crush trigger, in addition to the rate sensitivity of the constituent materials. An added complication, in gaining a deeper insight into crush, is borne out of the majority of studies which indicate that the post-test crush morphology at different strain rates is similar, and any variations are usually explained by the level of debris ejected from the tested specimens. In other words, changes in the failure mechanisms of specimens tested at different strain rates are not readily apparent. Jacob *et al*. [2] further point out that impact testing, where the impact speed is reduced from a maximum value to zero, is a more accurate representation of a crash event whereas quasi-static crushing is not a true crash representation since the tests are conducted at constant speed. Nonetheless, quasi-static testing permits the generation of strain rate data and is appropriate for computational modelling validation.

The implementation of strain rate sensitivity within an explicit numerical code is a straightforward matter. The challenge lies in ensuring that material characterization tests, used to generate strain rate sensitivity data, are not significantly influenced by specimen geometry and other structural factors, leading to the aforementioned lack of consensus.

#### (iv) Friction

The role of Mohr–Coulomb friction was discussed in §2d(ii) dealing with matrix-dominated intralaminar failure. Friction between delaminated surfaces should also be accounted for in a crush analysis as well as friction between the crash structure and the impacting surface. One of the advantages of computational modelling is that it permits the breakdown of the different energy-dissipating mechanisms during a crush simulation, providing insight into the prevailing damage mechanisms at play. Work carried out by the author and colleagues shows that friction can be a significant energy-dissipating mechanism even when no specific measures are taken to increase frictional forces between contacting surfaces [5,83,89]. In the specimens tested, approximately 15–30% of energy was dissipated through friction during crushing. Most of this energy dissipation was due to the friction generated between the crushing composite and the impacting surface. This is confirmed by separate modelling of a closely related structural response associated with post-impact compressive failure of a damaged structure, i.e. CAI strength [55]. In this modelling scenario, crushing of the composite was localized around the damage site, away from the supporting surfaces, leading to complete structural failure. The contribution of friction to energy dissipation was determined to be less than 5% of the total energy dissipation.

## 3. Material characterization

Ply-level material data are required for mesoscale modelling and well-established standards are available for the determination of basic material properties required for preliminary structural analysis, at quasi-static and dynamic loading. Additional testing is required to determine interlaminar and intralaminar static and dynamic fracture toughness values as input parameters for the computational model. This section highlights progress and challenges in the acquisition of this data. Laminate-level material characterization is also discussed.

### (a) Mode I interlaminar fracture toughness

For Mode I quasi-static fracture toughness tests, the ASTM double cantilever beam (DCB) standard is widely used [90] which generates an initiation and propagation value of fracture toughness. The initiation value is highly sensitive to the crack-tip conditions and the propagation value is a preferable input parameter for computational modelling. Strain rate testing of Mode I fracture toughness of UD carbon/epoxy composites was investigated by increasing the displacement rate of the DCB arms to up to 400 m s^−1^, of specimens mounted in a servo-hydraulic testing machine, where a reduction in fracture toughness was observed [91]. For higher strain rates, a split Hopkinson pressure bar (SHPB) was used to drive a wedge in a pre-existing crack of a mounted specimen at speeds of up to 1000 m s^−1^ where, in this case, the values obtained were nearly equal to the static values [92]. In the former approach, fibre bridging, which enhances the apparent fracture toughness, was shown to reduce with increasing load rate whereas the crack propagation in the latter tests was by means of driving a wedge in a pre-existing crack for a relatively short crack extension where such fibre bridging would not have been able to develop. Reduction in Mode I fracture toughness with strain rate for both thermoplastic (PEEK) and thermoset DCB carbon fibre composite specimens was also reported in [93].

### (b) Mode II interlaminar fracture toughness

An ASTM Mode II quasi-static fracture toughness test standard [94] based on a three-point end notch flexure (ENF) test is also available although this test only yields an initiation value. A four-point version of this test permits the determination of a propagation value [95] as crack growth is stable. Moderate increases in loading rates (from quasi-static up to 0.19 m s^−1^) did not reveal clear evidence of load rate dependence [96]. A modified cracked lap shear (CLS) Mode II test specimen was loaded at different rates in [97] and the crack velocity measured. It was shown that the velocity plateaued at a value well below the Rayleigh wave velocity and suggested that as more energy was supplied to the specimen, not all was being consumed in driving the predominant crack, but that energy was also being consumed in the creation of microcracks around this primary crack. In effect this suggested an increase in Mode II fracture toughness with increasing crack velocity.

### (c) Mixed-mode interlaminar fracture toughness

Mode I/Mode II mixed-mode fracture toughness testing makes use of a mixed-mode beam (MMB) bending test which generates stable crack growth over a range of mode mixity [98]. Dynamic mixed-mode tests, using different specimen geometries and conducted in either a SHPB or a servo-hydraulic test machine have yielded mixed results [99,100].

### (d) Longitudinal intralaminar fracture toughness

Longitudinal (loading in the fibre direction) intralaminar fracture toughness is an important parameter as the high specific energy absorption of carbon fibres governs the level of crashworthiness of a crush structure. Compact tension (CT) and compact compression (CC) tests, adapted from tests designed for metals [101,102], have been developed [103,104] with various modifications proposed to mitigate known problems of damage occurring at the loading points and areas of high stresses away from the crack tip [105,106]. An alternative approach which does not require the tracking of a propagating crack tip, as is required for CT/CC testing, is to exploit the size-effect law, originally proposed by Bazant & Planas [107], where a series of self-similar double-notched specimens of different sizes are tested in tension or compression and the results used to generate resistance (*R*-) curves [108–110]. Provided that CT/CC/size effect tests are conducted carefully, there is a high level of consistency in the results obtained under quasi-static loading conditions.

### (e) Transverse and shear intralaminar fracture toughness

Transverse tensile intralaminar fracture toughness may be assumed to be equal to the interlaminar Mode I fracture toughness discussed earlier [111]. For transverse compressive intralaminar fracture toughness, since the primary failure mode is similar to that observed under shear loading, this value may be assumed to be similar to the intralaminar matrix fracture toughness under shear loading. The V-notched rail shear test [112] was used by the author and colleagues [104] to determine the full nonlinear constitutive response, and the intralaminar shear fracture toughness determined by combining this test with the essential work of fracture approach [113].

### (f) Crushing stress

In the Introduction, the concept of a crushing stress was presented as a critical strength for a structure crushed against an impacting surface. In essence, all the damage mechanisms activated during crushing are encapsulated in a single variable. This level of homogenization permits macroscale modelling of crushing structures where elements within a crush zone are ‘consumed’, when this crush stress is reached, and removed from the analysis. This crush stress is determined experimentally and is considered a laminate property as opposed to an intrinsic material property of the composite used to construct the laminate. Hence different laminate lay-ups may require separate tests. Crush stress is also dependent on geometry where curved structures exhibit higher crush stress [25]. This is attributed to delamination suppression ahead of the crush zone which, presumably, permits the fibres to do more work by preventing splaying. This type of analysis is available as part of a commercial finite-element suite. The acquisition of quasi-static and dynamic crush stress data is based on the testing of flat coupons with a saw-tooth trigger using two anti-buckling fixtures, one of which emulates the suppression of delamination attributed to curvature [114].

This phenomenological model is used with single shell elements through the laminate thickness. A structural finite-element model will need to also account for the possibility of damage and/or failure occurring away from the crash zone. Hence, a damage model, separate from a crush stress, needs to also be activated to account for this possibility. In the commercially available implementation of this approach, an assumption is made that crush stress attributed to the finite-element model is binary, i.e. one value for flat geometry and another for curved geometry. It is readily apparent that this is a limitation.

Nonetheless, this macroscale approach provides a pragmatic multiscale crash modelling strategy. Mesoscale modelling may be used to simulate the laminate-level coupon tests which in turn require material-level data. A change in laminate lay-up will necessitate the execution of an additional mesoscale model of the coupon which is more straightforward than physically testing new specimens.

### (g) Comment

It is evident that despite concerted efforts to deduce the strain rate sensitivity of interlaminar fracture toughness of CFRP composite materials, this issue has yet to be satisfactorily resolved. This in turn poses a challenge to computational modelling. Nonetheless, the body of evidence suggests that under increasing strain rate, a reduction in Mode I fracture toughness is observed. This is supported by a study which showed that the fracture toughness of neat epoxy resin also decreased with increasing strain rate [115]. Weaker evidence of fracture toughness enhancement is reported for Mode II whereas for mixed-mode loading the situation remains unclear.

There seems to be better consensus in the literature suggesting an enhancement in intralaminar properties with strain rate. For example, detailed studies which focused on assessing the intralaminar strain rate sensitivity of UD CFRP materials using a SHPB indicated that even though at strain rates of around 100 s^−1^ the longitudinal modulus was not strain rate sensitive, an increase in compressive strength was observed [116]. The same group reported increases in transverse compression/shear modulus, yield and failure strengths for strain rates of approximately 350 s^−1^ [117]. Daniel *et al*. [118] showed that transverse and shear moduli and strength of a UD composite increased linearly with the logarithm of strain rate (from quasi-static to 1000 s^−1^). Leite *et al*. [119,120] reported an intralaminar longitudinal fracture toughness of CFRP material which increased linearly with strain rate under both tensile and compressive load over a range from quasi-static to 400 s^−1^.

Intermediate strain rate testing, often undertaken using a servo-hydraulic test machine, is sometimes hindered by dynamic interactions between the test machine and specimen [121]. Steps are required to mitigate this interaction, often through the use of damping materials at the load introduction mechanism.

## 4. Examples

The following three examples, in increasing complexity, demonstrate the state-of-the-art in crush modelling at the meso- and macroscale undertaken by the author and colleagues, using the computational model and strategies described herein.

### (a) Carbon fibre/epoxy UD/woven hybrid laminate crush specimen

**Figure 6.**
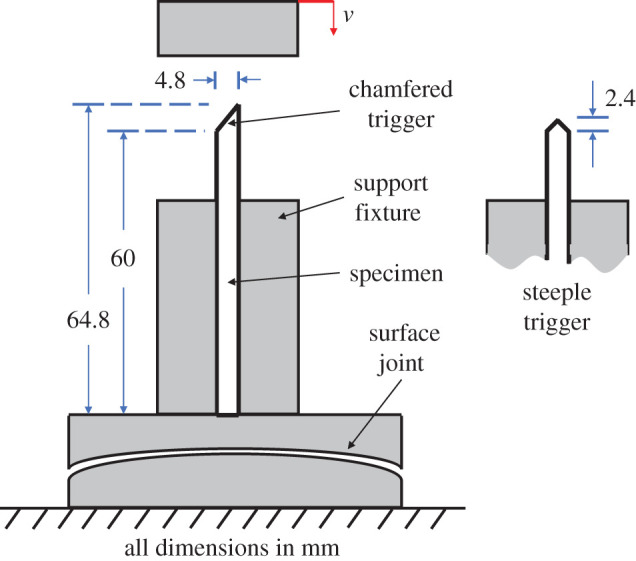
UD/woven hybrid specimen geometries and schematic of test fixture. (Online version in colour.)

A set of specimens with two types of triggers (chamfered and steeple) were prepared from a laminate with a five-harness satin (5HS) woven carbon fibre/epoxy composite on the outer surfaces and the rest UD CFRP plies [5HS/−45/+45/90/0/−45/+45/90/+45/−45/90/+45/−45/0/90/+45/−45/5HS]. Full details of this study are reported in [89] and only pertinent information is provided here. The geometry of these specimens, as mounted in the test fixture, is shown in figure 6, and these were crushed quasi-statically. The finite-element model used eight-node brick elements with single-point integration. An element size of 0.25 mm was used in the trigger region and 0.5 mm elsewhere. To ensure that accurate bending behaviour was captured, three elements through the ply thickness were used, resulting in a model with over 14 000 elements. Mass scaling, increases in loading rate, enhanced hourglass and distortion control were employed to stabilize the solution and execute within a reasonable time. Each simulation took approximately 16–20 h on a high-performance computer (HPC) cluster tocomplete.

**Figure 7.**
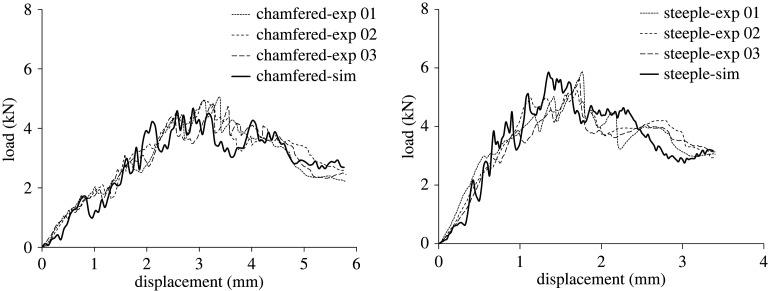
Load–displacement curves for chamfered-triggered and steeple-triggered UD/woven hybrid crush specimens.

**Figure 8.**
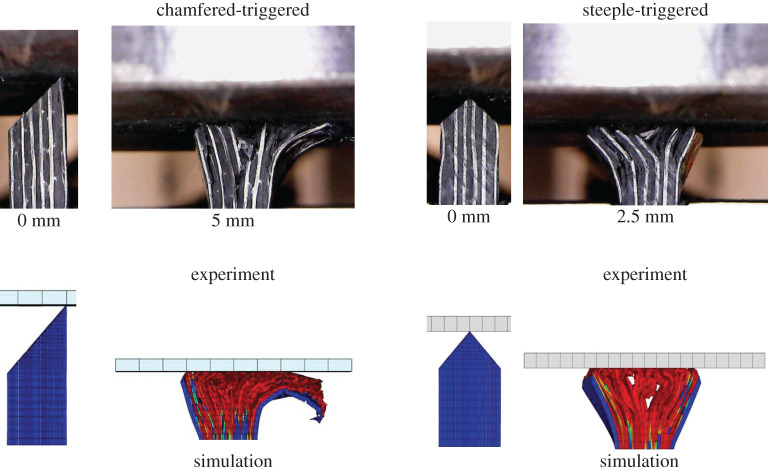
Damage morphology for both chamfered-triggered and steeple-triggered UD/woven hybrid specimens. (Online version in colour.)

The load–displacement response of the crushing specimens was captured with good accuracy as shown in figure 7. Figure 8 also shows good qualitative correlation between experimental and numerical damage for both specimen types.

### (b) Carbon fibre/thermoplastic corrugated laminate crush specimen

**Figure 9.**
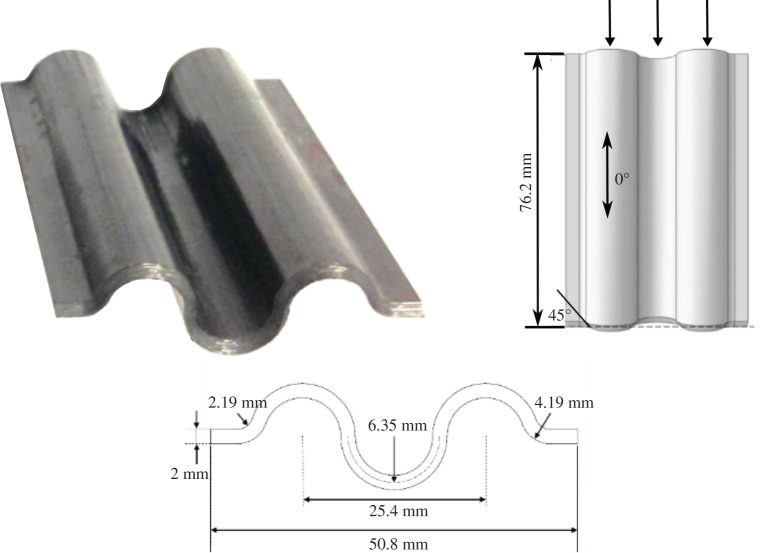
Carbon/fibre thermoplastic corrugated composite specimen geometry. (Online version in colour.)

**Figure 10.**
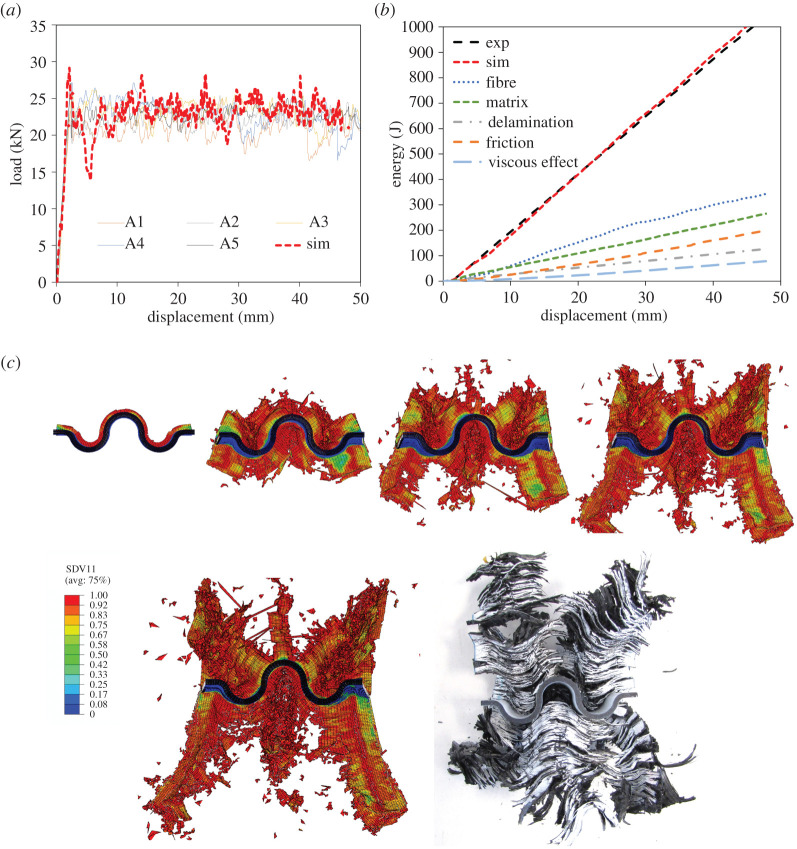
(*a*) Load–displacement and energy–displacement curves; (*b*) crush morphology for carbon/fibre thermoplastic corrugated composite specimen. (Online version in colour.)

In the study reported in [83] cross-ply corrugated chamfered specimens were manufactured from carbon fibre/thermoplastic UD composites. A self-supporting specimen is shown in figure 9 and all were crushed quasi-statically. A similar modelling approach to that described in §4a was implemented and took approximately twice as long to complete the analysis. With reference to figure 10, again it is noted that good qualitative and quantitative correlation was achieved.

### (c) Side impact structure of a Formula 1 car

**Figure 11.**
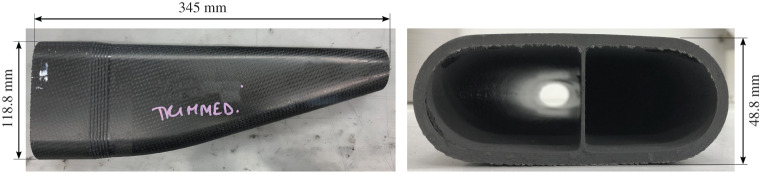
Side impact structure (SIS) geometry. (Online version in colour.)

A side impact structure (SIS) is a homologated crush tube common to all Formula 1 cars. There are two SISs mounted on each side of the car, an upper and lower one, each with a different lay-up. In the study detailed in [16], and discussed briefly here, the upper SIS was modelled and is shown in figure 11. This structure was made from 5HS woven CFRP and has a relatively complex geometry, with a varying cross-section along its crush length, different curvatures, ply drop-offs and an internal rib. Three SIS specimens were physically tested quasi-statically in a servo-hydraulic testing machine, and two dynamically, in a purpose-built crash-test facility at Cranfield University, using a sled impactor. In both cases, a high degree of consistency was achieved.

The primary aim of this work was to explore the measurement and use of representative crush stresses for macroscale modelling. Flat and cylindrical specimens with a lay-up corresponding to the crush section of the SIS were tested quasi-statically in a servo-hydraulic machine, and dynamically using a drop tower. For each geometry, a reduction in crush stress was observed with increased strain rate. These tests served to also highlight the care required in the acquisition of this data. For example, large oscillatory responses were obtained from the dynamically tested coupons which could be due to specimen/machine interactions. Unpublished work [122] was also undertaken to model the coupons quasi-statically using the mesoscale damage model, used in the previous two examples, and comparable crush stresses were obtained, indicating a route to practical multiscale analysis.

**Figure 12.**
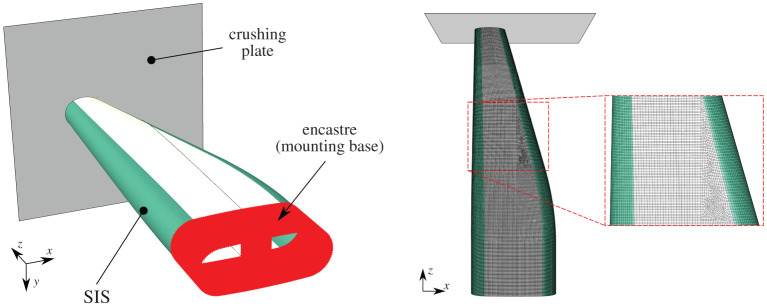
Finite-element model of side impact structure (SIS). (Online version in colour.)

The SIS finite-element model made use of 2D shell elements with single-point integration and one element through the thickness. The model, shown in figure 12, contained approximately 59 000 elements and quasi-static and dynamic crush stress values were used to model the crush zone.

**Figure 13.**
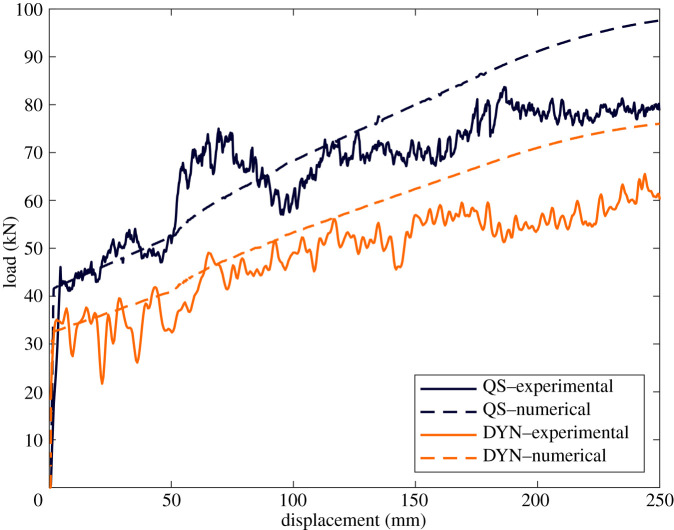
Load–displacement curves of F1 side impact structure (SIS). (Online version in colour.)

Figure 13 compares the numerical load–displacement curves with representative experimentally obtained quasi-static and dynamic curves. It is noted that a lower energy absorbing capacity was recorded in the dynamic tests, as was the case with the coupon tests. This reduction was attributed to an observed increase in delamination compared with the quasi-static tests. As discussed earlier, the formation of delamination dissipates less energy than damage associated with the fibre. An increase in delamination during dynamic testing is also consistent with material characterization tests described in §3 where the evidence points towards a reduction in Mode I interlaminar fracture toughness with strain rate as opposed to an apparent enhancement in longitudinal intralaminar fracture toughness.

It is noted that reasonable correlation was achieved although the numerical results tended to overestimate the load response later in the crash event. This may be due to several possibilities. Only two crush stress values were used which do not fully cover all the geometries present in the SIS. It may also be the case that the balance between failure modes dictating the crushing stress of the coupons may not be readily scalable. For example, large-scale delamination in the SIS, leading to a reduced energy absorbing capacity of the structures, may not be adequately represented at the coupon level.

## 5. Concluding remarks

The pursuit of ever lighter transportation structures has seen the increased utilization of carbon–fibre composite materials. Such structures are required to meet specific crashworthiness criteria and while the specific energy absorption of these materials can exceed that offered by conventional metallic structures, their effectiveness in a composite crush structure is highly dependent on the structure's geometry, fibre architecture, lay-up and initial crush conditions. Development of these structures through physical testing is expensive and time consuming and the use of computational modelling to reduce the extent of physical testing offers the means to lower these costs.

Computational modelling of crushing of carbon fibre-reinforced composite structures remains a challenging problem owing to the complex interacting failure mechanisms and the potential for numerical instability. These failure mechanisms initiate at the nano/microscale and evolve across scales to lead to macroscale crushing. In this overview, a high-fidelity mesoscale damage model was described which accounts for (i) interlaminar (delamination) failure in a laminated construction and (ii) intralaminar failure, characterized by fibre-dominated and matrix-dominated damage within a representative volume element of a ply. Continuum damage and fracture mechanics principals were used to represent the initiation and propagation of damage within each ply.

This damage model was incorporated within a commercially available explicit finite-element solver. Additional computational strategies were explained to stabilize the analysis. The computational cost of mesoscale modelling becomes prohibitive for modelling large structures and the use of a crush stress to encapsulate all the ensuing damage in a crush zone, in a single variable, was also explored.

The acquisition of accurate material data is crucial to the predictive capability of the numerical model. In particular, test methods for determining the quasi-static interlaminar fracture toughness values are becoming well established but the extent of strain rate influence has yet to be satisfactorily resolved for Mode II and mixed-mode fracture. Intralaminar fracture toughness values are more difficult to obtain but there seems to be stronger evidence of an apparent fracture toughness enhancement with strain rate. Hydraulic test machine/specimen interaction, which is particularly prevalent at intermediate strain rate testing, has been shown to hinder measurements in this range and steps should also be considered to minimize this effect.

It is evident that further progress can be made towards the development of a robust and efficient computational tool for crashworthiness assessments, along with improvements associated with test methods for the acquisition of the required material data. Nonetheless, a number of examples, presented in this paper, demonstrate that good correlation may be achieved between simulation and physical testing.

Future research in the field should continue to pursue the development of robust, reliable and computationally efficient multiscale modelling methodologies to ease the burden on industry in undertaking crashworthiness assessments of composite structures. Novel approaches to accurate material characterization under intermediate strain rates, which manage to resolve the conflicting data in the literature on strain rate dependence, would mark a step change in our understanding of composite material behaviour under dynamic loading.

## Data Availability

This article does not contain any additional data.
